# Fatty Acid Surfactant Photochemistry Results in New Particle Formation

**DOI:** 10.1038/s41598-017-12601-2

**Published:** 2017-10-04

**Authors:** Peter A. Alpert, Raluca Ciuraru, Stéphanie Rossignol, Monica Passananti, Liselotte Tinel, Sebastien Perrier, Yoan Dupart, Sarah S. Steimer, Markus Ammann, D. James Donaldson, Christian George

**Affiliations:** 1 Univ. Lyon, Université Claude Bernard Lyon 1, CNRS, UMR 5256, IRCELYON, Institut de recherches sur la catalyse et l’environnement de Lyon, 2 avenue Albert Einstein, F-69626 Villeurbanne, France; 2 Paul Scherrer Institute, Laboratory of Environmental Chemistry, 5232 Villigen, PSI Switzerland; 3 Department of Chemistry, University of Toronto, 80 Saint George Street, Toronto, Ontario, M5S 3H6 Canada; 4 UMR ECOSYS, INRA, AgroParisTech, Université Paris-Saclay, 78850 Thiverval- Grignon, France; 5 Present Address: Aix Marseille Université, CNRS, LCE UMR 7376, 13331 Marseille, France; 6 Present Address: Department of Physics, University of Helsinki, 00014 Helsinki, Finland; 7 Present Address: Wolfson Atmospheric Chemistry Laboratory, Department of Chemistry, University of York, York, YO10 5DD UK; 8 Present Address: Department of Chemistry, University of Cambridge, Cambridge, CB2 1EW United Kingdom

## Abstract

Organic interfaces that exist at the sea surface microlayer or as surfactant coatings on cloud droplets are highly concentrated and chemically distinct from the underlying bulk or overlying gas phase. Therefore, they may be potentially unique locations for chemical or photochemical reactions. Recently, photochemical production of volatile organic compounds (VOCs) was reported at a nonanoic acid interface however, subsequent secondary organic aerosol (SOA) particle production was incapable of being observed. We investigated SOA particle formation due to photochemical reactions occurring at an air-water interface in presence of model saturated long chain fatty acid and alcohol surfactants, nonanoic acid and nonanol, respectively. Ozonolysis of the gas phase photochemical products in the dark or under continued UV irradiation both resulted in nucleation and growth of SOA particles. Irradiation of nonanol did not yield detectable VOC or SOA production. Organic carbon functionalities of the SOA were probed using X-ray microspectroscopy and compared with other laboratory generated and field collected particles. Carbon-carbon double bonds were identified in the condensed phase which survived ozonolysis during new particle formation and growth. The implications of photochemical processes occurring at organic coated surfaces are discussed in the context of marine SOA particle atmospheric fluxes.

## Introduction

Aerosol particles reside in the atmosphere where they scatter and absorb light and impact the global radiative balance between the Earth and Sun, thereby influencing the climate^1–3^. A major component of aerosol particles is organic material, which can be directly emitted or formed *in situ* by secondary processes^4,5^. The latter contributes about 60 and 95% of organic aerosol mass in urban and rural air, respectively^6,7^, and significantly impacts global estimates of aerosol optical depth^8,9^. Predicting organic aerosol concentrations remains difficult, especially in marine environments due to a lack of observations and the possibility of unknown sources of volatile organic compounds (VOCs) as potential secondary organic aerosol (SOA) precursors^10^. It remains undecided if SOA particles pose a potential warming or cooling of the Earth and the uncertainty in their radiative forcing is comparable with other aerosol particle types i.e., sulfate, nitrate and biomass burning aerosols^9,11^. Although well recognized as important, SOA particles are not yet included when estimating anthropogenic radiative forcing changes between modern day and pre-industrial conditions due to an incomplete understanding of their formation^9^.

Our study suggests a new source of SOA particles due to the emission of VOCs photochemically produced by fatty acids at the air-water interface^12^. Organic surfactant compounds reside in the atmosphere on air-water interfaces such as surfaces of aqueous aerosol particles and hydrometeors^13–20^. A concentrated layer of organic and biogenic material also resides at the sea surface, referred to as the sea surface microlayer (SML); this includes surfactants, lipids, proteins, polysaccharides, fatty acids, alcohols, and fragments of cellular materials, all of which can be aerosolized and have been detected on marine derived airborne particles^21–28^. Recent laboratory studies have linked aerosol production^29^ and fatty acids composition (8–18 carbon atoms per molecule)^27^ with marine biological activity supporting the notion that aerosol bound surfactants from ocean waters may be pervasive. When irradiated by sunlight, surfactants on water, aerosol particles and droplets may undergo unique photochemical reactions primarily due to the distinctly different physical and chemical properties of interfaces compared with the underlying bulk or overlying gas phases^12,30^.

Photochemical reactions at organic interfaces have received increasing attention, as they are suspected to affect atmospheric trace gas and aerosol budgets^12,31–36^. Photosensitized reactions, in particular, occur when chromophores such as humic acids or chlorophyll absorb light and then react with neighboring molecules. They are important under conditions where direct photolysis of organic compounds, nitrogen oxide or O_3_ is not efficient. Light can enhance the loss of gas phase oxidants such as O_3_ to chlorophyll in seawater, resulting in production of other radicals such as OH and Cl^33,35^. In the presence of the photosensitizer imidazole-2-carboxaldehyde (IC) and light, oxidation of limonene was observed which has implications for photosensitized SOA formation in aqueous aerosol^36^ and production of HO_2_ radicals^37^. Recent work details evidence that photosensitized chemistry between IC and octanol under irradiation occurs at the organic-coated aqueous interface and results in the production of unsaturated carbon bonds and carbonyl and carboxyl functionalities^34^. Production of saturated and unsaturated VOCs was observed due to photosensitized reactions at the air-water interface of a fatty acid and humic acid solution^32^. Subsequent ozonolysis of these compounds led to new particle formation and growth^31^.

Photoactivity of organic surfactants without the presence of a previously known photosensitizer, i.e. IC, was recently observed and could be explained by a triplet state excitation from a spin forbidden electronic transition^12^. Experiments were performed in a flow reactor with a short residence time on the order of seconds and thus, observations of SOA nucleation which typically occurs on longer time scales was not possible^12^. Although a new source of SOA particles due to fatty acid photochemistry was suggested, observational evidence and evaluation of its importance to aerosol nucleation was lacking.

Here, an investigation of SOA particle formation due to photochemistry occurring at surfactant interfaces is presented. Experiments were conducted in a multiphase atmospheric simulation chamber^31^, in which a filled basin of water resided with one of two organic surfactants, nonanoic acid or nonanol. Concentrations of VOCs over time were monitored while the interface was illuminated with UV radiation having a wavelength range similar to that of UV light from the sun hitting the Earth’s surface. The nucleation and subsequent growth of SOA particles in the chamber was observed following dark ozonolysis reactions initiated immediately after UV lights were switched off. Additional experiments were performed to investigate UV light exposure and simultaneous UV and ozone exposure on SOA particle production. The organic carbon composition of the SOA was quantified with discrimination of various functionalities including unsaturations, hydroxyl, carbonyl and carboxyl functionalities using scanning transmission X-ray microscopy coupled with near-edge X-ray absorption fine structure spectroscopy (STXM/NEXAFS). A comparison between measured carbon spectral signatures of SOA particles formed in our chamber and previous laboratory and field studies is presented. The atmospheric implications of our results are discussed in the context of particle production rates and fluxes derived from experimental data.

## Photochemical production of nanoparticles above a surfactant coated interface

**Figure 1:**
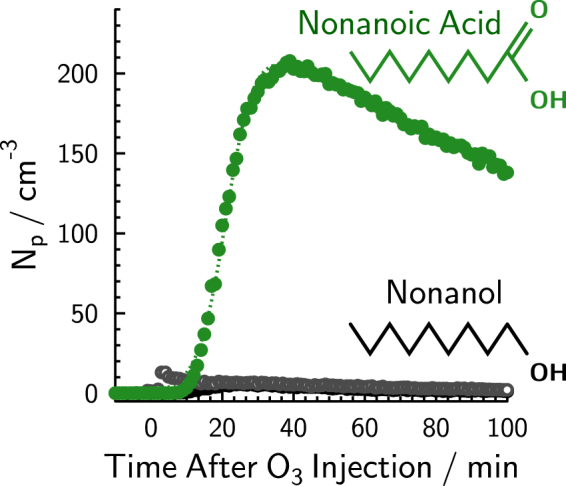
Concentration of aerosol particles having diameters > 3 nm, *N*_p_, in the chamber as a function of time, *t*, for two different experiments using either nonanoic acid or nonanol (shown as green or black symbols, respectively). Grey symbols indicate a single experiment using pure water for comparison (these data are reproduced as the blue symbols in Fig. S11). At *t* < 0, the chamber was irradiated with UV light. At t = 0, irradiation stopped and ozone was injected into the chamber. The dotted green line is the result from a sigmoidal fit given in the text.

Figure 1 shows particle concentration, *N*_p_, in the atmospheric simulation chamber over time, *t*, for an experiment in which nonanoic acid at an air-water interface inside the chamber was illuminated with UV light followed by the introduction of gas phase ozone in the dark. Nucleation of SOA particles up to a maximum concentration, *N*_p,max_ ~200 cm^−3^ was observed after ozone was introduced. New particle formation did not occur after chamber irradiation when nonanol was used as a surfactant or when only water was present as shown in Fig. 1. The UV irradiation spectrum (Fig. S1) and full detailed experimental results are presented in the Supplementary Information. Background SOA particle concentrations were always <10 cm^−3^ in experiments, as outlined in the Supplementary Information. SOA particle formation above 10 cm^−3^ was considered significant and observed for all irradiation followed by ozone exposure experiments using nonanoic acid (Figs S2–S5) as the surfactant where *N*_p,max_ ranged from 14–2600 cm^−3^. After two hours, particles grew to around 100 nm in diameter, presumably due to continued reaction and condensation of low volatility ozonolysis products. For the results presented in Fig. 1, irradiation by UV light stopped when ozone was injected into the chamber to ensure dark ozonolysis of the photochemically produced VOCs. Therefore, we conclude that photochemistry of the fatty acid led to the production of unsaturated products^12,38^ which then reacted with ozone ultimately leading to SOA formation.

In a different experiment shown in Fig. S6, a mixed aqueous solution of humic acid and nonanoic acid was used in the chamber, resulting in SOA particle formation with *N*_p,max_ on the order of 10^4^ cm^−3^. Previous work has shown that the presence of the humic acid photosensitizer enhances photochemical reactions and thus, enhances SOA formation^31^. However, the results shown in Fig. 1 clearly demonstrate that the presence of a photosensitizer is not a requirement for fatty acid photochemistry to proceed. In fact, our results strongly suggest that SOA particle nucleation and growth as seen in Fig. 1 was due entirely to unsaturated gas phase SOA precursors photochemically produced at a nonanoic acid interface^12^.

Figure 1 also shows results of an experiment using nonanol, a C_9_ alcoholic surfactant, during which background levels at *N*_p_ < 10 cm^−3^ of air were maintained despite UV irradiation and ozone introduction (further details of this experiment can be found in Fig. S7 of the Supplementary Information). This result compares with a recent report showing that photochemical reactions could take place at an octanol (a C_8_ alcoholic surfactant) interface, but only in the presence of a photosensitizing compound^34^. In accord with our results, the authors concluded that octanol alone does not photochemically react when exposed to UV light^34^.

In the absence of a photosensitizer we found that photochemistry of nonanol did not occur while the corresponding C_9_ carboxylic acid was, in fact, photoactive. Rossignol *et al*.^12^ discussed several possibilities for why the nonanoic acid-coated water surface shows photoreactivity, whereas the alcohol-coated surface does not. Our observations point to a specific role of the terminating functional group of the surfactant for possible photochemical SOA formation pathways at an air-water interface i.e., the acid group may play a role in photochemical properties of surfactant molecules. Neat fatty acid liquids can undergo singlet-triplet state excitation centered at 272 nm^12,39^ however, the reason why this absorption band is present at high concentrations and not observed for single molecules (e.g. in the gas phase) remains unknown. We speculate that fatty acids undergo unique intermolecular interactions when closely spaced, i.e. at a highly concentrated interface. Shorter chain fatty acids, C1–C4, can form stabilized hydrogen bonded dimers in solution with a parallel orientation, in which the distance between carbon atoms of two monomer head groups is 5–6 Å^40^. The surface pressure of a nonanoic acid monolayer is approximately 35 dynes cm^−1^ measured at about 1 mM concentration^41^ which corresponds to a range of 5–10 Å^2^ per molecule^42^, a closer head group distance (~3 Å) than expected for dimer formation in solution. This may influence the hydrogen bonding network and delocalize carbonyl bonds in nonanoic acid giving rise to >300 nm UV light absorption. In turn, absorption can allow for triplet state excitation, which is accessible at high concentrations of the acid, i.e. in the neat liquid^12^.

**Figure 2:**
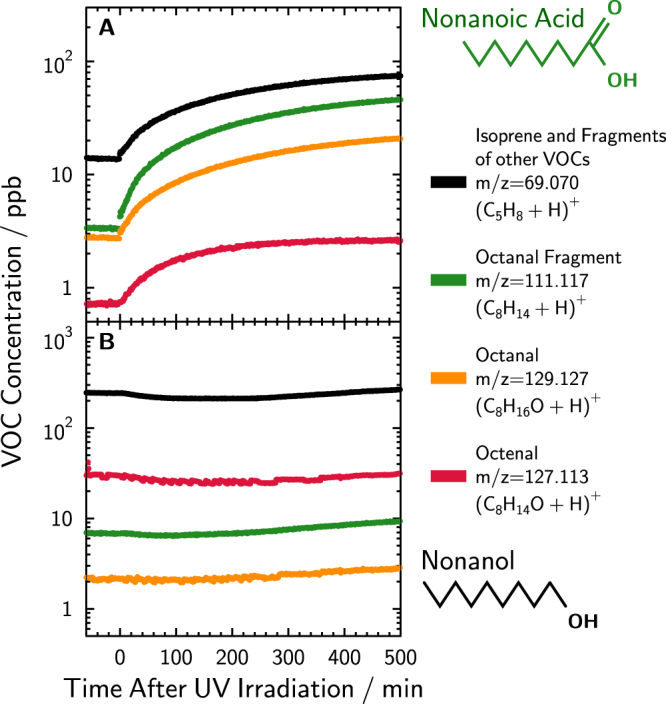
Concentration of volatile organic compounds (VOCs) as a function of time, *t*, for two different experiments using either (**A**) nonanoic acid or (**B**) nonanol measured from proton transfer reaction with H_3_O^+^ reagent ions. At t = 0, UV irradiation of the chamber and basin started. Saturated and unsaturated gas phase compounds and corresponding detected atomic mass to charge ratios, m/z, are indicated.

The formation of particles can be explained by the photochemical production of VOCs in the chamber via the mechanism proposed in Rossignol *et al*.^12^, followed by reaction with ozone yielding SOA particles. The concentrations of selected VOCs measured in the chamber using PTR-MS detection are shown in Fig. 2A and B for the two surfactants nonanoic acid and nonanol, respectively. Two of the measured gas phase compounds that underwent proton transfer reaction with H_3_O^+^ reagent ions shown are octenal (C_8_H_14_O detected at *m/z* = 127.11) and octanal (C_8_H_16_O detected at *m/z* = 129.13), which are unsaturated and saturated aldehydes, respectively. Also shown is C_8_H_15_^+^ (detected at *m/z* = 111.12), a common fragment of octanal^38^ and C_5_H_9_^+^ (detected at *m/z* = 69.07) having contributions due to isoprene, (C_5_H_8_ + H)^+^, and fragmentation products of other parent ions^32^. These VOCs and other detected alkanes, alkenes, and saturated and unsaturated aldehydes were identified using H_3_O^+^ and NO^+^ reagent ions^38^. These same compounds were previously shown to be prominent photochemical products in solution^12^. Figure 2A shows that, in fact, unsaturated aldehydes were present in the chamber prior to SOA formation and their production exactly started when UV illumination began. Note that in Fig. 2, UV irradiation starts at t = 0. Clearly, photochemical production of VOCs continued over time and eventually reached a steady state concentration when using nonanoic acid (Fig. 2A). The production of VOCs as a function of UV irradiation was also investigated. We observed that increasing the number of UV lamps used for irradiation increased the VOC concentration in the chamber (Figs S2 and S3). Therefore, greater UV intensity yielded greater VOC production and emission.

**Figure 3:**
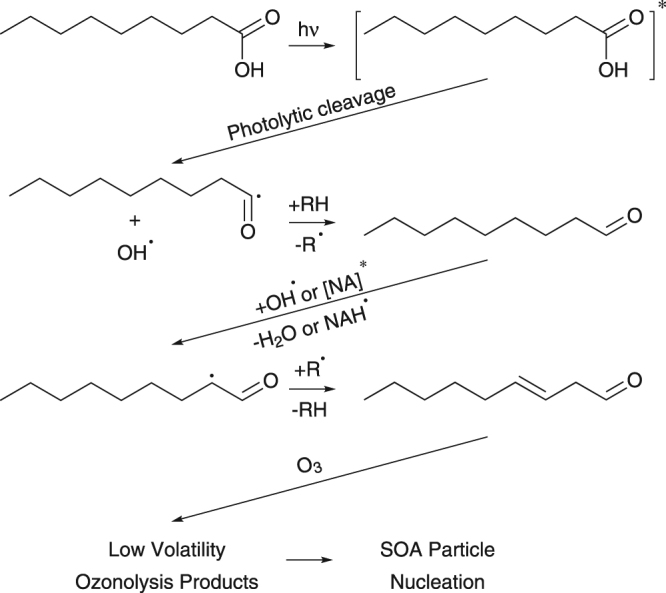
Photochemical reaction mechanism from Rossignol *et al*.^12^ including ozonolysis of products resulting in SOA formation.

A reaction mechanism for VOC production and SOA formation is shown in Fig. 3. In the initial step, nonanoic acid is excited to a triplet state upon UV light absorption. Rossignol *et al*.^12^ proposed that photolytic cleavage is one mechanism among others leading to the formation of radicals, including in this case the OH radical. Subsequent reactions with neighboring organic molecules should result in aldehyde formation. The presence of OH and excited nonanoic acid can result in further radical chemistry, unsaturated aldehyde formation and H_2_O elimination, the latter being favorable in the organic rich layer. Reactions between unsaturated compounds and O_3_ results in products with low volatility capable of SOA particle nucleation as previously discussed. Figure 2 includes concentrations of 4 VOCs produced in the chamber; however, a great variety of other unsaturated aldehydes, unsaturated acids, alkenes and dienes have been observed as well. Therefore, we suspect that there is not just a single SOA precursor compound, but that these all may act as precursors.

Experiments were performed to evaluate the role of an air-water interface on nonanoic acid surfactant photochemistry by injecting gas phase nonanoic acid into the chamber without the presence of water in the basin (Figs S8 and S9). Nonanoic acid is expected to partition to the chamber walls resulting in a surface concentration there. If nonanoic acid was present on the Teflon chamber walls with sufficient surface coverage, UV irradiation of this organic interface may have resulted in photochemical production of SOA precursor gases. This may be feasible if the molecular surface coverage of nonanoic acid, *A*_na_, on the Teflon walls was on the order of 5–10 Å^2^ as was present at the air-water interface^41,42^. The vapor pressure of nonanoic acid is on the order of 2 ppm at 25 °C (~14 µg m^−3^)^43^, which is about 3–5 times higher than observed in chamber experiments where gas phase nonanoic acid concentrations ranged 0.5–0.8 ppm. To our knowledge, estimates of nonanoic acid molecular surface adsorption and *A*_na_ on Teflon films at subsaturated conditions have not been made. Previous investigations of oxidized organic vapor partitioning on Teflon walls was done by Zhang *et al*.^44^, however their formulation is driven by vapor concentrations exceeding supersaturation, which is not the case in our experiments. Previous research has shown that time scales for organic monoacids to equilibrate with Teflon chamber walls occurs on the order of minutes^45^. Therefore, our experimental time scales on the order of 1000 min between introduction of surfactants and UV irradiation should have allowed for adsorption-desorption equilibrium conditions, such as *A*_na_ or wall concentration, *C*_w_, to be met. To estimate *A*_na_, we first assume that nonanoic acid partitioned to Teflon walls following Henry’s law as a rough estimate. This assumption neglects that other organic molecules (in addition to water) may be present on the walls due to the previous experimental chamber history, although at concentrations which we have shown to be negligible for measurements of SOA or VOC production. For an observed nonanoic acid partial pressure of 8.1 × 10^−2^ Pa and a Henry’s law constant^46^, *H* = 6.9 × 10^−3^ mol L^−1^ Pa^−1^, *C*_w_ = 5.6 × 10^−4^ mol L^−1^. Following Seidl^41^, *C*_w_ equates to a spreading pressure of about 27 mN m^−1^. Finally, the pressure-area isotherm for nonanoic acid measured by Gilman *et al*.^42^ yields *A*_na_ = 9 Å^2^ for our conditions which is slightly greater than what is calculated for the water surface as previously discussed. Therefore, a surface layer of nonanoic acid on the walls at these concentrations may have been responsible for photochemical VOC production. We note that these estimates are uncertain and thus, more investigation must be done to better understand organic vapor wall partitioning and the effect on surfactant photochemical reactions.

When nonanol was used as a model surfactant, UV irradiation did not yield any VOC production and instead, concentrations were perturbed toward decreasing values as seen in Fig. 2B. We also performed UV irradiation of nonanol in presence of ozone in an attempt to induce OH chemistry and VOC production, however we again observed a decrease in VOC concentrations. This implies that photochemical radical formation was not sufficient to initiate degradation of the surfactants and production of SOA precursors. We note that nonanoic acid and nonanol can fragment as a result of ionization *via* proton transfer reaction and may contribute to different background VOCs detection prior to UV illumination. Concentrations of VOCs shown in Fig. 2 were not background subtracted for completeness. We conclude that SOA nucleation and growth was not possible due to lack of photochemically produced VOCs from the surfactant nonanol.

## Presence of particle phase organic functionalities and carbon double bonding

**Figure 4:**
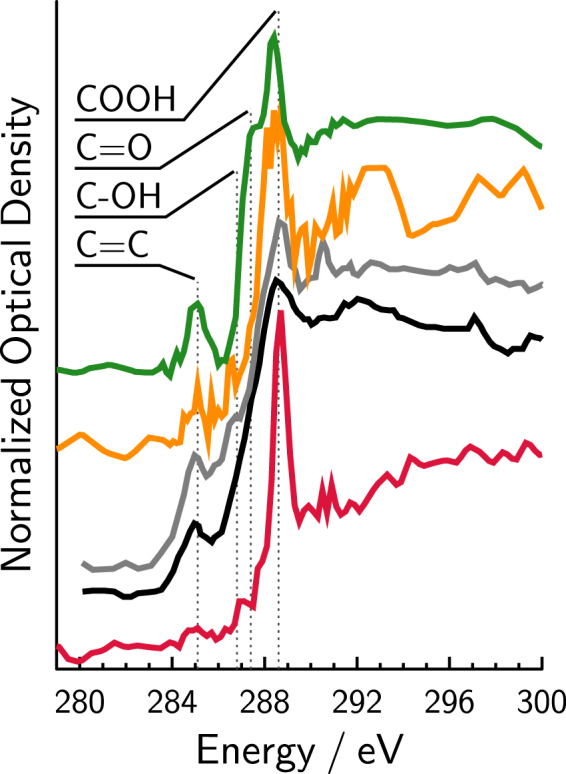
Average X-ray absorption spectra of secondary organic aerosol (SOA) particles generated from 2 different experiments in which i) nonanoic acid, humic acid and water was irradiated with UV light followed by dark ozonolysis (green, average of 8 particles) and ii) nonanoic acid and water was irradiated with UV light followed by ozonolysis with continued UV light exposure (orange, average of 11 particles). Field collected particles from Takahama *et al*.^49^ referred to as “type-i” and “type-j” are shown as black and grey curves, respectively. The spectrum of stearic acid shown as the red curve is a reference for saturated fatty acid material. Possible peaks due to various carbon functionalities are indicated as vertical dotted lines for carboxyl (COOH), carbonyl (C=O), hydroxyl (C-OH) and carbon double bonds (C=C) at 288.6, 287.4, 286.8 and 285.1 eV, respectively.

The chemical composition of single SOA particles collected from two different chamber experiments were investigated using STXM/NEXAFS. Figure 4 shows X-ray absorption spectra from two types of SOA particles formed: i) under simultaneous ozone and UV light exposure using only the surfactant nonanoic acid and water (orange spectrum) and ii) under dark ozonolysis subsequent to UV exposure of nonanoic acid and water containing humic acids (green spectrum). Unfortunately, it was impossible to investigate NEXAFS spectroscopy of particles formed by dark ozonolysis experiments with nonanoic acid (Fig. 1) only because particle deposition was too sparse. Spectra from multiple particles were normalized to the difference in optical density at 300 and 280 eV and averaged to compare spectral shape of the different SOA particle types. We note that particle to particle variability was similar.

Figure 4 shows an absorption peak at 285.1 eV indicating that unsaturated carbon bonds are present in the SOA particles investigated here. We note that this feature is much stronger for the humic acid experiment (green), which generated significantly more particle density than for the non-humic acid experiment (orange), and that formation of aromatic compounds due to nonanoic acid photochemistry was ruled out in previous studies^12^. The presence of carbon double bonds is surprising since ozonolysis should have depleted any carbon unsaturation in VOCs produced in our experiments. Ozonolysis of VOCs ultimately led to the formation of condensed phase oxidized compounds observed as absorption peaks in Fig. 4. Measured spectra of both particle types exhibited oxidized functionalities, including the carboxyl, carbonyl and hydroxyl functional groups evident from absorption peaks at 288.6, 287.4 and 286.8 eV, respectively^47^. As expected, the main feature of the X-ray absorption spectra of a reference fatty acid, stearic acid (red spectrum), was a peak observed at 288.6 eV due to the carboxyl functionality, but lacking other oxygenated functional groups. It may be possible that continued heterogeneous reaction of ozone or OH in the presence of UV light with SOA particle surfaces yielded aged compounds with carbon double bonds. Alternatively, we suggest that oxidized VOCs with different volatilities initially having multiple carbon unsaturations partitioned to the particle phase with carbon double bonds that survived ozonolysis. The latter explanation points toward increasing evidence for the photochemical production of unsaturated compounds at the interface^38^. Once in the condensed phase, these organic functionalities and unsaturated compounds may be buried by continual condensation of SOA and protected from surface O_3_ reaction surviving for long time scales^48^.

Absorption spectra of field collected particles from a variety of sources, including marine, terrestrial and urban were reported in Takahama *et al*.^49^ and used here for comparison with chamber-formed SOA particles. Among those reported, the most similar spectra are of particles referred to as “type i” and “type j” characterized as multiple-transition aerosol particles, i.e. particle exhibiting a variety of carbon functionalities^49^. The spectral shapes of the field collected and chamber-formed SOA particles are qualitatively similar, i.e. the main feature is the carboxyl functionality, a secondary C=C peak and a broad absorption shoulder occurring between 286.0-288.2 eV, indicating hydroxyl and carbonyl functionalities. Takahama *et al*. suspected a variety of potential compounds exhibit these features, such as humic and fulvic acids^50^ and others stemming from soil and biomass burning sources^47^, however these particular features were seen in every marine, terrestrial and polluted environment investigated^49^. We suggest that these highly functionalized particles found in the atmosphere by Takahama *et al*.^49^ may have a contribution due to secondary photochemical sources stemming from SOA precursors such as those produced from photochemical reactions at a surfactant interface.

## Atmospheric Implications

From the observations described above, surfactant fatty acid photochemistry may impact the budget of SOA particles in the atmosphere, and especially at low wind speeds over the oceans where the organic and surfactant rich SML is irradiated by sunlight. While our experimental design allows an understanding of the photochemical process at the air-water interface, it nevertheless cannot catch the full complexity of marine boundary layer processes. Nevertheless, we attempt to estimate the potential importance of SOA formation from our results.

**Figure 5:**
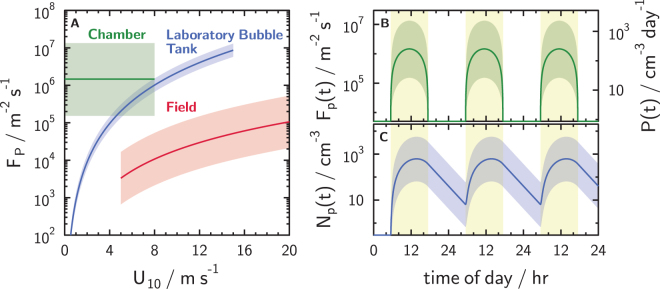
**(A)** Flux of secondary organic aerosol (SOA) particles, *F*_P_, as a function of the 10-m high wind speed, U_10_, derived from aerosol particle production rates in chamber experiments. The range of average *F*_P_ values over a 45 min new particle formation event are shown as the green shading where the geometric mean is given by the solid line. For comparison, primary emitted marine aerosol particle flux formulations from previous literature are shown as blue and red curves indicating parameterization from a laboratory study by Salter *et al*.^52^ and from field measurements derived using wet and dry deposition methods by Lewis and Schwartz^51^, respectively. Lines indicate mean values with shadings representing uncertainties. (**B**) Simulated diurnal flux, *F*_P_(*t*), particle production rates, *P*(*t*), and (**C**) concentrations of SOA particles, *N*_P_(*t*), as a function of time. Solid lines and shading indicate the mean and range of values, respectively, as a function of time over three days. Yellow shading indicates daytime hours. See text for more information.

Particle concentrations over time, *N*_p_(*t*), for all chamber experiments were reproduced as a continuous function by fitting to an exponentially decaying sigmoidal equation,

1 $${{\boldsymbol{N}}}_{p}(t)=\frac{{{\boldsymbol{b}}}_{1}{{\boldsymbol{e}}}^{-{\boldsymbol{kt}}}}{1+{{\boldsymbol{e}}}^{-{{\boldsymbol{b}}}_{2}({\boldsymbol{t}}-{{\boldsymbol{b}}}_{3})}},$$

where *b*_1_, *b*_2_ and *b*_3_ are fitting constants and *k* = 6.2 × 10^−3^ min^−1^ is the particle loss rate independently determined from the decay of *N*_p_ over time after nucleation was over. Losses were due to chamber walls and the sampling air flow out of the chamber. This sigmoidal function was arbitrarily chosen to capture the initial increase in *N*_p_ as a function of *t*, the change in concavity as *N*_p_ approached maximum values and the decay of *N*_p_ after new particle formation stopped. Figure 1 shows the resulting fit for *b*_1_ = 260 cm^−3^, *b*_2_ = 0.25 min^−1^ and *b*_3_ = 21.5 min. Particle production rates, *P*(*t*), were then determined by

2 $$\frac{{\rm{\Delta }}{\boldsymbol{N}}({\boldsymbol{t}})}{{\rm{\Delta }}{\boldsymbol{t}}}={\boldsymbol{P}}({\boldsymbol{t}})-{\boldsymbol{k}}{{\boldsymbol{N}}}_{p}({\boldsymbol{t}})$$

using the known particle loss rate, *kN*_p_(t), and the time rate of change of particle concentrations, Δ*N*_p_(*t*)/Δ*t*, calculated from Eq (1). A corresponding aerosol particle flux, *F*_p_(*t*) was derived as

3 $${{\boldsymbol{F}}}_{p}({\boldsymbol{t}})={\boldsymbol{P}}({\boldsymbol{t}})\frac{{{\boldsymbol{V}}}_{{\rm{ch}}}}{{{\boldsymbol{A}}}_{{\rm{int}}}}r{\boldsymbol{,}}$$

where *P*(*t*) is multiplied by the ratio between the chamber volume, *V*_ch_, the air-water interfacial area, *A*_int_, and finally by *r* = 11.3, which is the ratio between the integrated solar irradiance (91.7 W m^−2^) and chamber irradiance (8.1 W m^−2^) at 300–420 nm in wavelength assuming particle production is linear in irradiance. Average *F*_p_ values from all experiments employing nonanoic acid at the air-water interface (Figs S2–S5) were calculated over a 40 min period corresponding to the duration of SOA particle nucleation and growth events. Figure 5 presents our derived *F*_p_ = 1.47 × 10^6^ (particles) m^−2^ s^−1^ with an order of magnitude uncertainty determined from the maximum and minimum average *F*_p_ values over time (see Fig. S10 in the Supplementary Information).

In addition to our derived flux of SOA particles, Fig. 5a also shows flux parameterizations of primary emitted marine aerosol particles, also known as sea spray aerosol (SSA) particles^51,52^ for comparison. It has been suggested that full coverage of a SML is maintained up to a wind speed, *U*_10_, on the order of 8 m s^−1^ measured at a 10 m height^53^, and so we assume our derived SOA particle flux is constant as function of wind speed. Total flux of SSA particles reported in Lewis and Schwartz^51^ from field measurements using multiple wet and dry deposition flux derivation methods are between 7 × 10^2^–5 × 10^5^ m^−2^ s^−1^ depending on *U*_10_. Values of *F*_p_ from Salter *et al*.^52^ who used an air bubble entrainment method to generate SSA are about 1–2 orders of magnitude greater than Lewis and Schwartz^51^ for the same *U*_10_ range. It appears that, *F*_p_ derived from our chamber experiments would possibly point toward a significant contribution of particles under low wind speed conditions. As previously discussed, if nonanoic acid was present on the Teflon chamber walls at concentrations similar to that of the air water interface, then similar photochemical processes may have occurred there. Then flux calculations, Eq (3), should employ *A*_int_ equal to the total internal chamber surface area ~10 m^2^, which would decrease our reported flux values by a little over 1 order of magnitude. We do underline that the chamber fluxes are not derived from real marine conditions and do not consider i) losses of oxidized VOCs to chamber walls which tends toward under prediction of SOA formation, ii) that high ozone concentrations not typical for the atmosphere were employed in experiments, iii) experimental variability in relative humidity and temperature, iv) that pH and surface tension values are not within a range of ambient oceanic conditions, v) that seed aerosol or pre-existing particles in ambient air will scavenge VOCs and vi) that only pure water was employed. Nevertheless, our data do suggest that further attention should be given to marine surface photochemistry.

This can also be highlighted by simulating the aerosol particle concentrations in a column of air with a height of 0.5 km (representing a column integrated boundary layer), to investigate the potential importance of our experimentally derived flux on marine aerosol particle concentrations following de Leeuw *et al*.^54^. We assume that extrapolated SOA particle flux scales with a 12-hour diurnal solar irradiance cycle at the Earth’s surface (Fig. 5b) i.e., *F*_p_(*t*) = −*F*_p_°sin(π*t*/12 + 8), where *F*_p_° = 1.47 × 10^6^ m^−2^ s^−1^ with an uncertainty taken from Fig. 5a. Simulated *F*_p_(*t*) values and corresponding particle production rates, *P*(*t*), are shown in Fig. 5b. When assuming a particle turnover time of 3 days^54^, simulated aerosol particle concentrations, *N*_p_(t), shown in Fig. 5c reach about 600 cm^−3^ on average with an uncertainty range between about 60–6000 cm^−3^ during the daytime hours. During the night when *F*_p_ = 0 m^−2^ s^−1^, *N*_p_ decreases over time. By comparison, aerosol particle concentrations in clean marine regions typically are <300 cm^−3^ ^51,54^.

This discussion indicates that SOA formation due to fatty acid photochemistry at the SML can play a potentially significant role in determining total aerosol particle concentrations in the marine boundary layer. Many soluble, insoluble and particulate organic components reside at the SML and are concentrated with respect to bulk ocean water. Carboxyl functionalities which can be common in marine organic matter are suggested to be photochemically active. If so, the SML may be a location for photochemical reactions similar to what has been observed here resulting in an increase in VOC and SOA particle loading in the boundary layer. In turn, surfactant photochemical SOA particle production may be an additional source of particulate organic matter, which can help close the gap for calculating organic aerosol concentrations that are consistently under-predicted in marine environments^10^. We note that this is an abiotic photochemical source of SOA precursors as opposed to direct emissions of biogenic SOA precursor gases. Increasing particle concentrations and particle sizes due to photochemical SOA formation may also impact atmospheric budgets of cloud condensation nuclei, which are especially important in clean marine environments where cloud forming particles can be relatively scarce compared with polluted environments. Certainly, future studies should aim to better constrain estimates and uncertainties of this abiotic photochemical SOA formation pathway for atmospheric application.

## Conclusions

We demonstrated SOA nucleation and growth due to dark ozonolysis of unsaturated organic gas phase products arising from photochemistry at a nonanoic acid-coated aqueous interface. Although the potential for SOA production was suggested in previous studies, limited chamber volumes and short residence times were not optimal for detecting new particle formation. The SOA produced had unique carbon bonding signatures and include carbon unsaturation and oxygenated functionalities. The X-ray spectra measured here may be used as a reference for comparison against other laboratory and field collected organic aerosol probed with the same technique. We have found that a nonanoic acid surface may be necessary to explain photochemical production of VOCs in our experiments, but this can be either an air-water or air-solid interface. Intermolecular interactions may occur at a nonanoic acid surfactant interface due to the fact that nonanoic acid molecules are so highly concentrated there. Thus, light absorption and photochemistry may be altered compared to purely gas phase processes, which can explain observed VOCs and subsequent SOA formation. The surfactant nonanol did not yield any photochemical products under the same conditions as experiments using nonanoic acid highlighting the potential influence of closely spaced acid groups at an interface on light absorption. Therefore, photochemistry at interfaces in the presence of saturated fatty acids of various carbon chain lengths or other organic components with carboxyl functions may also result in saturated and unsaturated VOCs and SOA precursors. Fatty acids and other organic surfactants and materials from a variety of sources are frequently observed not only in the sea surface microlayer, but also in marine and terrestrial aerosol particles. Cloud droplets are known to possess surfactant compounds, and photochemistry occurring on their surface may therefore be a source of SOA precursors. We have shown that SOA particle nucleation can occur even under high relative humidity (~75%). Previous studies have observed ultra-fine and accumulation mode particle concentrations within and at the borders of clouds which may have been the result of new particle formation over spatial scales of 100 m^55–57^. This new surfactant photochemical process may also then contribute to in-cloud SOA nucleation. We suggest that interfacial organic photochemistry is ubiquitous, occurs in a variety of environments under a wide range of conditions, results in the emission of VOCs and SOA nucleation and growth and constitutes a condensed phase radical source important for organic aerosol and trace gas budgets affecting the oxidizing capacity of the atmosphere and air quality.

## Methods

Experiments were conducted in a 2 m^3^ multiphase atmospheric simulation chamber following Bernard *et al*.^31^. Note that the chamber used here has been significantly improved over the study by Bernard *et al*.^31^ by lowering its particle background levels, thereby increasing its sensitivity to new particle formation. A basin holding 30 L of water (resistivity of 18.2 MΩ cm) was placed at the bottom of the Teflon chamber to which nonanoic acid (97%, 70 Alfa Aesar) or nonanol (99% Alfa Aesar) was added at 2 mM resulting in a pH of about 4^32^. These covered the 0.6 m^2^ water surface in excess such that surfactant lenses were in equilibrium with a monolayer at its respective equilibrium spreading pressure^32^. When desired, humic acid was added to the water at a concentration of 3 mg L^−1^. Visible and UV fluorescent light irradiated the chamber volume and the surfactant at the air-water interface^31^. Figure S1 shows the photon flux measured with a calibrated spectrophotometer^58^. Gas phase concentration of volatile organic compounds (VOCs) was monitored with a selective reagent ion time of flight mass spectrometer (SRI-ToF-MS 8000, Ionicon Analytik) using either H_3_O^+^ or NO^+^ as source reagent ions. For all experiments (Figs S2–S9), settings for the instrument source of the SRI-ToF-MS 8000 were optimized following Rossignol *et al*.^12^ and Tinel *et al*.^38^. In the H_3_O^+^ mode, VOCs undergo proton transfer reaction yielding protonated parent ions using a 4.6 mA source current, 116 V source voltage, 84 V source outlet voltage and 5.8 sccm flow of water vapor. These settings resulted in O_2_^+^ and NO^+^ source impurities at <5% of the H_3_O^+^ signal. Despite optimization, high relative humidity (RH) up to 80% experienced in the chamber yielded (H_2_O)H_3_O^+^ ion clusters amounting to about 20% of total ions. In the NO^+^ mode, reactions with VOCs undergo charge transfer, hydride ion transfer, hydroxide ion transfer or clustering. Instrument settings used in NO^+^ mode were 5.5 mA source current, 24 V source voltage, 70 V source outlet voltage and a 5.4 and 2.8 sccm O_2_ and N_2_ flow, respectively. These settings yielded total source impurities O_2_^+^, NO_2_^+^ and H_3_O^+^ at <10% of the reagent ion signal. For both ionization modes, drift tube pressure, temperature and overall applied voltage was 2.24 mbar, 60 °C and 600 V, respectively, which led to an E/N ratio of 135 Td (Townsend, 1 Td = 10–17 cm^2^ V^−1^) chosen to minimize the formation of water clusters due to high RH^38^. Temperature and RH in the chamber were recorded (Vaisala HMP110) and the concentrations of NO and NO_2_ (Eco Physics CLD88p coupled with PLC860) and O_3_ (Thermo Scientific 49i) were measured. Chamber experiments were performed in a N_2_ atmosphere with a slight positive operating differential pressure >3.5 Pa using a flow of ~7.5 L min^−1^ to compensate a total sampling flow of ~4.5 L min^−1^ and any chamber leaks. Ozone was generated using O_2_ gas at 0.5 L min^−1^ which passed either through a corona discharge or a UV light generator (Jelight Model 600) and then injected into the chamber.

The experimental chamber and water basin was scrubbed using ethanol, then rinsed with water and dried thoroughly before each experiment. After cleaning, the chamber was flushed with 40 L min^−1^ of N_2_ for more than 48 hours after which RH <5% was maintained. After flushing, background checks were performed using a 7.5 L min^−1^ flow of N_2_ and turning on and off visible and UV light and with and without the presence of 0.6–5.0 ppm of O_3_. Clean chamber conditions were met if particle concentrations remained below 1 cm^−3^ and the sum of NO and NO_2_ concentrations (NO_x_) was maintained at <0.6 ppb. After cleaning, 30 L of water was introduced into the basin at a liquid flow rate of ~1 L min^−1^ using a peristaltic pump and Teflon plumbing lines^31^. Background checks were again performed using UV irradiation and ozone injection into a chamber before and after filling the basin with pure water. Concentration of NO_x_ always remained <1 ppb and particle concentrations <10 cm^−3^ during water injection. After all background levels were established, the basin was emptied, dried and then refilled while injecting 10.5 mL of nonanoic acid inline using a syringe through a septum installed immediately before the basin^31^. The chamber was then flushed (40 L min^−1^ N_2_) for hours or days and returned to experimental conditions (7.5 L min^−1^ N_2_) for another day before commencing UV irradiation of the surfactant interface. When VOC concentrations reached an approximate steady state concentration while irradiated with UV, ozone was injected in a short burst (between 20 s and 2.5 min depending on the generator used) reaching a maximum chamber concentration of about 600 ppb. Before and after ozone injection, aerosol particle concentrations, *N*_p_, with particle diameters *d*_p_ > 3 nm were monitored (TSI, CPC 3776) and aerosol particle size distributions for *d*_p_ > 10 nm were measured (TSI, EC 3080 with a Long DMA and CPC 3772).

Figure S11 shows an example of *N*_p_ as a function of time after ozone injection in a chamber experiment having the lowest total observed particle numbers. The new particle formation event is clear from the steady increase of *N*_p_ that reached a maximum after 40 min, similar in time to other SOA particle nucleation events from experiments presented here. An example of an instance in which particle concentrations sharply increased immediately after ozone injection in a chamber containing pure water is shown as blue symbols. Particles inadvertently introduced into the chamber were likely due to contamination from the corona discharge ozone generator. Small particles were removed prior to entering the chamber by directing the O_2_/O_3_ flow through 10 m of ¼ inch Teflon tubing and 2 HEPA filters. Despite these measures, instances of particle contamination did occur, although infrequently and easily identifiable. These were not considered as new particle formation events. Particle contamination using the UV light generator did not occur.

The absolute irradiance (Fig. S1) was measured with a calibrated spectrophotometer probe^58^ located at a fixed point at the center of the chamber 10 cm above the air-water interface. Light produced from the UV fluorescent tubes used in chamber experiments largely had wavelengths, λ, between 300 to 400 nm. We note that measurements for λ < 300 nm yielded detection limit values on the order of 10^−3^ W m^−2^ nm^−1^, thus we assume that total light output in this range is negligible, but shown in Fig. S1 for completeness. In comparison, the solar spectrum at the Earth’s surface is shown derived using the online Quick Tropospheric Ultraviolet and Visible (TUV) calculator for a solar zenith angle of 0° (available at http://cprm.acom.ucar.edu/Models/ TUV/Interactive_TUV/). Rossignol *et al*.^12^ irradiated a 15 mL reactor cell half filled with a solution of 2 mM nonanoic acid and water using a Xenon lamp with an irradiance spectrum shown in Fig. S1. Although the light produced from the Xenon lamp was much more intense compared to the chamber, especially for λ < 300 nm, VOCs photochemically produced in their experiments were similar to those identified in chamber experiments presented here.

STXM/NEXAFS was used to investigate the organic carbon composition of SOA particles by irradiating them with X-rays and measuring absorption with high energy resolution. Nucleated SOA particles were collected on copper TEM grids with a quantifoil film (Agar Scientific) following R’mili *et al*.^59^ and brought to the PolLux beamline (X07DA) of the Swiss Light Source located at the Paul Scherrer Institute for analysis using STXM/NEXAXS^60^. A more detailed overview of this technique can also be found in previous literature^47,49^ and is only briefly described here. STXM/NEXAFS was used to explore the organic carbon functionality of particles using monochromatic X-rays with energy between 278–320 eV, which is the range of electron binding energies for ground state electron orbitals of the carbon atom, also known as the carbon K-edge. Absorption spectra of X-ray light at the carbon K-edge were converted to optical density (OD) as a function of X-ray energy. Energy calibration was performed by comparing the measured lowest energy peak of polystyrene with its literature value^61^. Series of particle OD images at closely spaced energy steps were taken with a spatial resolution of 35 × 35 nm. Reported spectra used a 2 ms pixel dwell time scanning over the entire particle and imaging at 98 energies or energy points. The OD averaged over the 2-D projected particle area is reported, where OD = −ln(*I*/*I*o), and *I* and *I*o are the transmitted and initial light intensities, respectively.

If particles were damaged by the X-ray beam, then it would be expected that the carbon absorption spectra would change significantly upon repeat exposure of the same particle. Figure S12 shows the spectra of the same particles taken three consecutive times each with a low resolution of 34 energy points, i.e. 34 X-ray images between 278–320 eV. Three consecutive acquired spectra at low energy resolution represent a low, normal and high X-ray exposure totaling 34, 68 and 103 energy points, respectively. Note, that the spectra averaged in Fig. 4 were constructed from 68 energy points once using higher energy resolution, and therefore are similar in X-ray exposure as the “normal” exposure at low energy resolution. Despite the different exposures, the spectra remain similar thus experiencing no beam damage. Therefore, the spectra reported here were not altered by the X-ray beam and indicate actual carbon functionalities in condensed phase.

## Electronic supplementary material

Supplementary Information
