# Supplementary material for: Fatty Acid Surfactant Photochemistry Results in New Particle Formation
Source: Sci Rep. 2017 Oct 4;7:12693. doi: 10.1038/s41598-017-12601-2 (PMC5627235; doi:10.1038/s41598-017-12601-2)
Supplement: Supplementary file 1 — Supplementary Information [file 41598_2017_12601_MOESM1_ESM.pdf]

Supplementary Information for "Fatty acid Surfactant Photochemistry Results in New Particle Formation"

Peter A. Alpert[1,2,*] Raluca Ciuraru[1,a], Stéphanie Rossignol[1,b], Monica Passananti[1,c], Liselotte Tinel[1,d], Sebastien Perrier[1], Yoan Dupart[1], Sarah S. Steimer[2,e], Markus Ammann[2], D. James Donaldson[3] and Christian George[1,*]

[1]*Univ. Lyon, Université Claude Bernard Lyon 1, CNRS, UMR 5256, IRCELYON, Institut de recherches sur la catalyse et l'environnement de Lyon, 2 avenue Albert Einstein, F-69626 Villeurbanne, France.*

[2]*Paul Scherrer Institute, Laboratory of Environmental Chemistry, 5232 Villigen PSI, Switzerland.*

[3]*Department of Chemistry, University of Toronto, 80 Saint George Street, Toronto, Ontario M5S 3H6, Canada.*

[a]now at UMR ECOSYS, INRA, AgroParisTech, Université Paris-Saclay, 78850, Thiverval- Grignon, France.

[b]now at Aix Marseille Université, CNRS, LCE UMR 7376, 13331, Marseille, France.

[c]now at Department of Physics, University of Helsinki, 00014 Helsinki, Finland.

[d]now at Wolfson Atmospheric Chemistry Laboratory, Department of Chemistry, University of York, YO10 5DD, UK.

[e]now at Department of Chemistry, University of Cambridge, Cambridge, CB2 1EW, United Kingdom.

Detailed experimental results

Nonanoic Acid on a Pure Water Surface

Descriptions of all experiments reported here are summarized below and in Table 1. Figures S2-S5 are described below and relate to conditions in which nonanoic acid was present at the air-water interface. Two experiments were designed to elucidate the role

of UV light on the photochemical formation of VOCs and SOA particle nucleation and growth. Figure S2 shows background concentrations of VOCs without the presence of nonanoic acid in the chamber and under UV irradiation using 2-12 UV fluorescent tubes for t=500-950 min. Irradiation of the nonanoic acid surfactant interface at t=1950-2400 min results in increasing VOC concentration significantly above background levels. When nonanoic acid was injected in the chamber prior at t~1000 min, particle concentrations remained <10 cm^{-3}. In this particular experiment the CPC failed prior to ozone injection. N_p derived using the SMPS is shown as crosses in Fig. S2. After the SOA nucleation event, the CPC was repaired and continued to measure particle concentration in agreement with the SMPS. Figure S3 shows an experiment in which 6 and 12 lamps were used for illumination consecutively. When only 6 lamps were used, VOC production and steady state concentrations were less than when using 12 lamps. Thus, increasing light intensity implies increasing photochemical production of VOCs. UV illumination stopped when ozone was injected into the chamber to ensure that dark ozonolysis of unsaturated VOCs was responsible for SOA particle nucleation. Note that the time between tick marks in the right and left panel of A) are not the same. After SOA particle nucleation continued for 17 minutes, UV lights were switched on once again. Aerosol particles grew to detectable sizes about 25 min after ozone injection. When switching on and off lights, the temperature in the chamber would increase and decrease, respectively. To minimize temperature differences as much as possible, visible light fluorescent tubes were used when UV fluorescent tubes were not. In other words, whenever a certain number of UV tubes were switched on or off, the same number of visible light tubes were switched off

or on, respectively. In this way, temperature variations did not exceed 4° C in range. Figure S4 shows another experiment using nonanoic acid at the air-water interface using 12 UV lamps simultaneously switched on. Aerosol nucleation occurred while UV illumination was sustained. Despite the combination of UV light, ozone, humidity and thus OH radicals, aerosol nucleation resulted in maximum concentration of 14 cm^{-3} which is very close, but above to background values. This is the lowest aerosol particle concentration observed for all experiments, although the reason for this is not entirely clear. Figure S5 shows yet another experiment that again reveals photochemically produced VOCs due to UV irradiation of nonanoic acid resulting in aerosol particle nucleation from dark ozonolysis reaction. Aerosol particle concentrations reached a moderate value of about 200 cm^{-3} in this experiment.

Nonanoic Acid on the Surface of a Humic Acid-Water Solution

Figure S6 details an experiment in which nonanoic acid was at an air-aqueous solution interface of humic acid and water. It can be expected that in the presence of a photosensitizer, such as humic acid, enhanced VOC and aerosol production would be observed.[1] Similar VOCs were produced, and concentrations of the selected VOC's shown were not significantly greater than seen in Figs. S2-S5. However, aerosol particle nucleation due to dark ozonolysis resulted in particle concentrations that exceeded those for experiments without humic acids. Bernard et al. (2016)[1] found that the presence of a photosensitizer enhanced formation of SOA particles in agreement with our findings, but did not simultaneously observe VOC concentrations. Previous

investigations of SOA particle production, yield and volatility have been performed using mixtures of aromatic and aliphatic precursors which always resulted in greater aerosol yields and lower volatility, implying that a greater diversity of VOC's may result in enhanced SOA formation[2]. Alternatively, VOC species actually observed in our chamber experiments may not be those that control nucleation and particle number, and possibly humic acid triplet induced chemistry may lead to more of these nucleating agents. We suspect that photosensitized chemical reactions, as opposed to photochemical reactions, may result in a greater diversity of VOCs and thus greater aerosol production as seen in Fig. S6. Investigation and analysis are being done to elucidate the diversity of VOC's from chamber experiments in future studies.

Nonanol on a Pure Water Surface

Figure S7 shows experimental results when nonanol was used as a model surfactant at an air-water interface. When irradiated with UV light, concentrations of VOCs previously identified for experiments using nonanoic acid were not enhanced beyond their background levels. In a more detailed analysis not shown here, no enhancement of any peak was observed in the mass spectrum over time during UV irradiation. Ozone was introduced 3 times through the course of this experiment. UV illumination stopped simultaneously upon the first ozone injection and aerosol particle concentrations increased although remained at values $<10\ cm^{-3}$. The second and third ozone injection did not result in aerosol particle formation. From these results, we conclude that a

nonanol surfactant cannot participate in photochemical reactions and does not result in SOA particle nucleation.

UV Irradiation of Gas Phase Nonanoic Acid Without a Water Interface

Experiments were performed to evaluate the role of an air-water interface on nonanoic acid surfactant photochemistry by injecting gas phase nonanoic acid into the chamber without the presence of water in the basin. Figures S8 and S9 shows that despite the lack of an air-water interface, VOC products were still produced with steady state concentrations similar to experiments with nonanoic acid at an air-water interface. When only gas phase nonanoic acid was desired N_2 gas was passed into a 2L bottle containing 0.2 L of neat nonanoic acid heated to 30 ^{o}C. Nonanoic acid evaporated in the bottle and the flow was directed to the chamber resulting in a gas phase nonanoic acid concentrations similar to previous experiments. UV irradiation produced similar VOCs at concentrations consistent with experiments in which nonanoic acid was at an air-water interface. A small amount of liquid water (~100 mL) was injected into the chamber to provide humidity which gradually decreased from about 50 % at t=0 min to completely dry condition at t=5000 min (see Table 1). This change in relative humidity over time did not affect the production of VOCs in this experiment. During the experiment, the corona discharge ozone generator failed and a low power backup UV lamp generator was used instead. This resulted in a maximum ozone concentration of only 55 ppb, an order of magnitude less than in other experiments. Figure S9 shows an experiment under dry conditions where gas phase nonanoic acid was also introduced to the chamber. The

N_2/nonanoic acid flow was stopped during the middle of the experiment and replaced with a N_2 flow at about t=3100 min during which UV irradiation continued. Despite the decrease in the nonanoic acid concentration over 16 hours, concentrations of octanal and known octanal fragments were maintained at their enhanced levels. In contrast, the concentration of octenal decreased for decreasing nonanoic acid concentration under UV irradiation (t=3100-4000 min). In spite of this, when nonanoic acid concentration was low (t>4500 min), UV irradiation still led to VOC production.

Saturated and unsaturated VOC's produced from nonanoic acid photochemistry observed here is in agreement with recent experiments conducted with nonanoic acid at an air-water interface in a small (<15 mL) quartz photochemical reaction cell.[3] The authors observed that aqueous phase photochemistry was insufficient to explain their results, and directly determined that an air-water interface was necessary for photochemical reactions.[3] There, a high concentration of nonanoic acid is present compared to the underlying bulk and overlying gas phase. We suggest that intermolecular interactions and high concentrations may be required to explain our observed SOA formation. We note that solid surfaces may also exhibit high organic concentrations, and thus surfactant photochemistry of fatty acids that proceeds at an air-water interface may also proceed at an air-solid interface. We hypothesize that this would explain why VOCs were produced in the absence of water in our chamber experiments.

Flux and simulation of SOA particles in the atmosphere

Figure S10 shows the average and max and min values of particle flux, F_p, as a function of t from chamber experiments shown in Figs. S2-S5. Particle concentrations over time, $N_p(t)$, were fitted and used to derive particle production rates, $P(t)$, used in Eq. 3 to ultimate calculate F_p(t). Mean, maximum and minimum F_p(t) shown as the dark green line in Fig. S10 were used to derive estimates shown in Fig. 5.

References

1 Bernard, F., Ciuraru, R., Boréave, A. & George, C. Photosensitized Formation of Secondary Organic Aerosols Above the Air/Water Interface. *Environ. Sci. Technol.* **50**, 8678-8686, doi:10.1021/acs.est.6b03520 (2016).

2 Emanuelsson, E. U. *et al.* Formation of Anthropogenic Secondary Organic Aerosol (SOA) and its Influence on Biogenic SOA Properties. *Atmos. Chem. Phys.* **13**, 2837-2855, doi:10.5194/acp-13-2837-2013 (2013).

3 Rossignol, S. *et al.* Atmospheric Photochemistry at a Fatty Acid-Coated Air-Water Interface. *Science* **353**, 699-702, doi:10.1126/science.aaf3617 (2016).

4 NCAR. *Quick TUV Calculator*, <http://cprm.acom.ucar.edu/Models/TUV/Interactive_TUV/> (

Tables:

Table S1: Summary of experimental ionization mode of the SRI-ToF-MS, temperature, *T*, relative humidity, RH, and maximum particle concentrations N_{max}. Check marks indicate experiments where VOC or SOA production was observed and when dark ozonolysis was employed. Ozone generator type, corona discharge (CD) of UV lamp (L), is also indicated.

	Description	Ion Mode	VOCs Produced	Dark Ozonolysis	T / °C	RH / %	N_{max} / cm^{-3}	Nucleation event	Ozone Generator
**Fig. S2**	Nonanoic acid and water	H_3O^+, NO^+	√	-	27.6	72.4	2.7×10^3	√	CD
**Fig. S3**	Nonanoic acid and water	NO^+	√	√	27.1	77.5	1.2×10^2	√	CD
**Fig. S4**	Nonanoic acid and water	H_3O^+	√	-	28.6	73.9	1.4×10^1	√	CD
**Fig. S5**	Nonanoic acid and water	H_3O^+	√	√	26.3	77.7	2.1×10^2	√	L
**Fig. S6**	Nonanoic acid and humic acid-water aqueous solution	H_3O^+	√	√	27.0	71.5	9.3×10^3	√	L
**Fig. S7**	Nonanol and water	H_3O^+	-	√	30.2	64.6	$<10^1$	-	L
**Fig. S8**	Gas phase nonanoic acid	H_3O^+	√	√	29.4	0 to 48.6	$<10^1$	-	L
**Fig. S9**	Gas phase nonanoic acid	H_3O^+	√	-	31.9	< 1	$<10^1$	-	-

Figures:

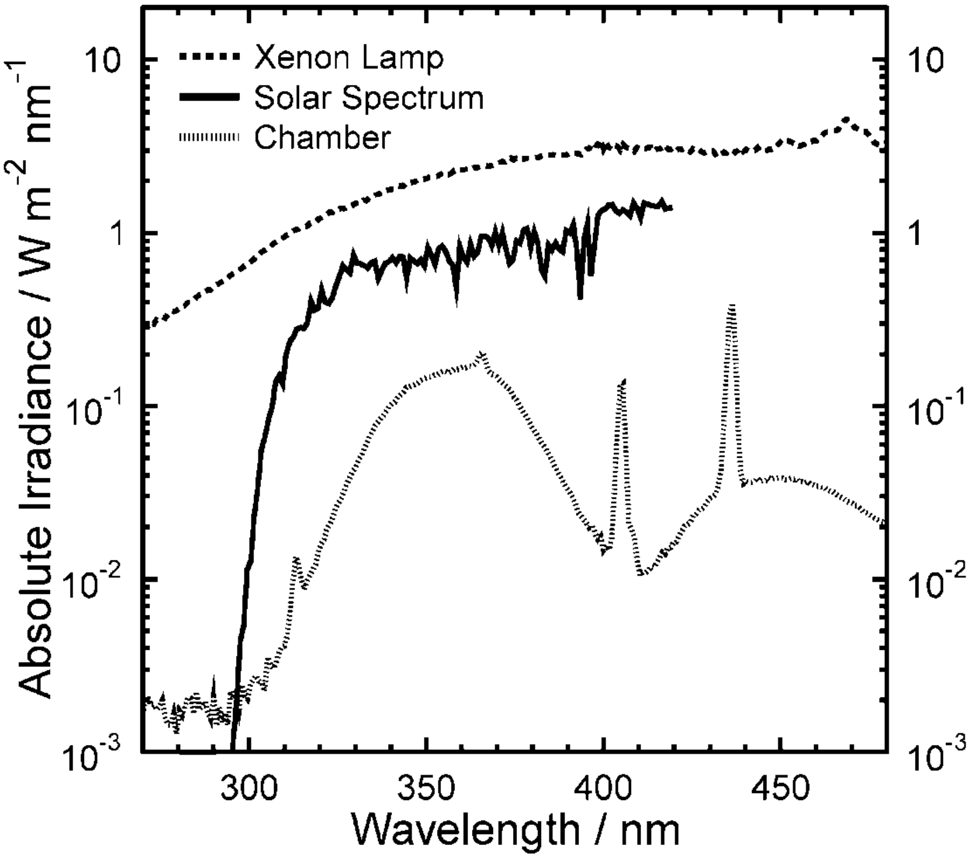

Figure S2: Absolute irradiance and a function of wavelength for the 12 UV fluorescent light tubes used in chamber experiments, the solar spectrum at the Earth's surface (TUV calculator[4]) and a xenon lamp[3] shown as dotted, solid and dashed lines, respectively.

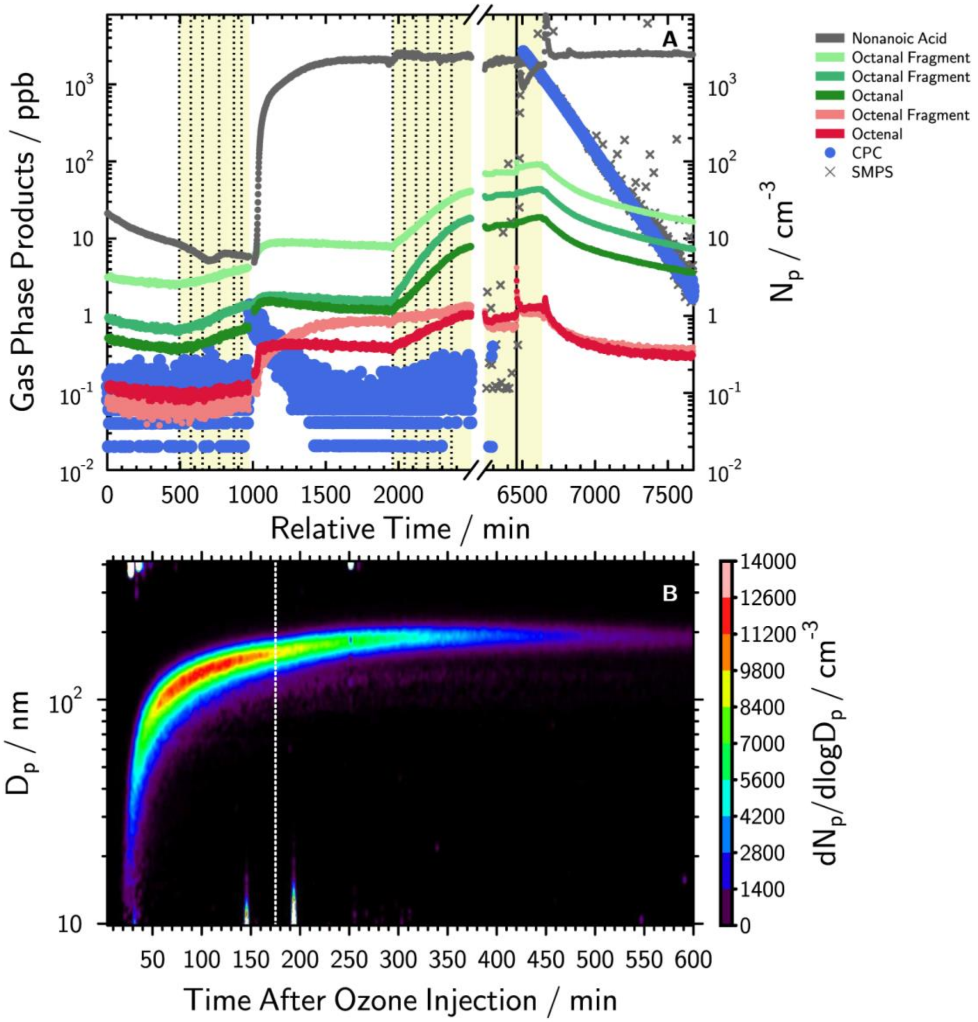

Figure S2: A) Concentration of volatile organic compounds (VOCs) observed using the H_3O^+ ionization mode and secondary organic aerosol (SOA) particles in a chamber experiment as a function of time, *t*, in which nonanoic acid was present at an air-water interface. Green, red, and grey colors indicated in the legend are selected VOCs and known fragments. Particle concentrations, N_p, measured using an ultra-fine condensation particle counter (CPC) and scanning mobility particle sizer spectrometer (SMPS) are also shown. UV illumination is indicated by the yellow shading. Vertical dotted lines indicate the time 2 additional UV fluorescent tubes were switched on, i.e. the 6 vertical lines in a row for t=500-950 min and t=1950-2400 min indicate 2, 4, 6, 8, 10 and 12 tubes consecutively switched on for illumination. The vertical solid line indicates the time when ozone was injected into the chamber up to 600 ppb in concentration. B) SOA particle size distribution, $dN_p/d\log D_p$, as a function of *t* after ozone injection for the nucleation event shown in panel A). The vertical dotted white line indicates the time when UV lights were switched off.

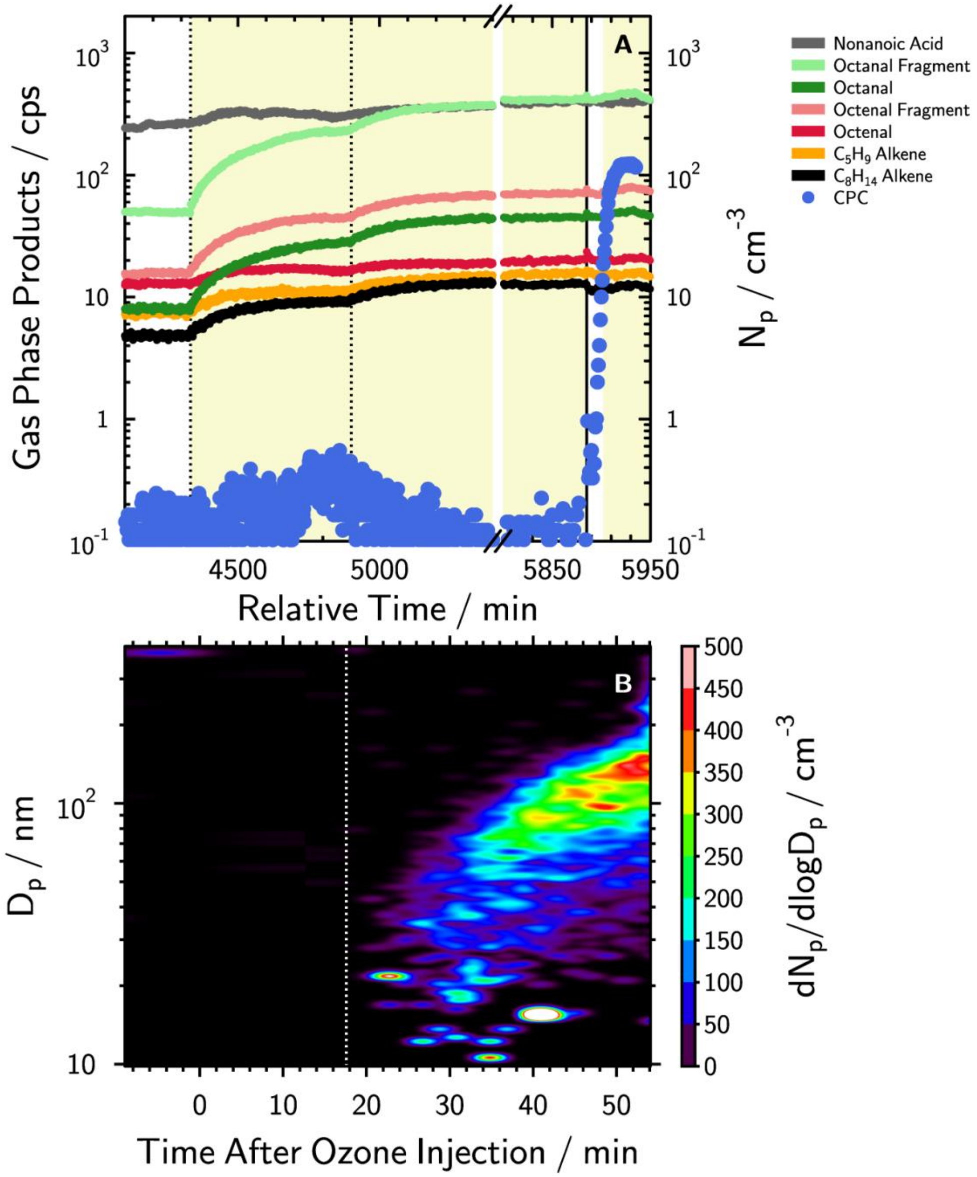

Figure S3: A) Concentration of volatile organic compounds (VOCs) using the NO^+ ionization mode in units of ion counts per second (cps) and secondary organic aerosol (SOA) particles in a chamber experiment as a function of time, *t* in which nonanoic acid was present at an air-water interface. Vertical dotted lines indicate the time which 6 additional UV florescent tubes were switched on, i.e. the 2 vertical lines for t=4300 and 4800 min indicate 6 and 12 tubes switched on for illumination. Note that the time between tick marks for the right and left panels of A) are not the same. B) SOA particle size distribution, $dN_p/d\log D_p$, as a function of *t* after ozone injection for the nucleation event shown in panel A). The vertical dotted white line indicates the time when UV lights were switched on. All other lines and shadings are the same as in Fig. S2.

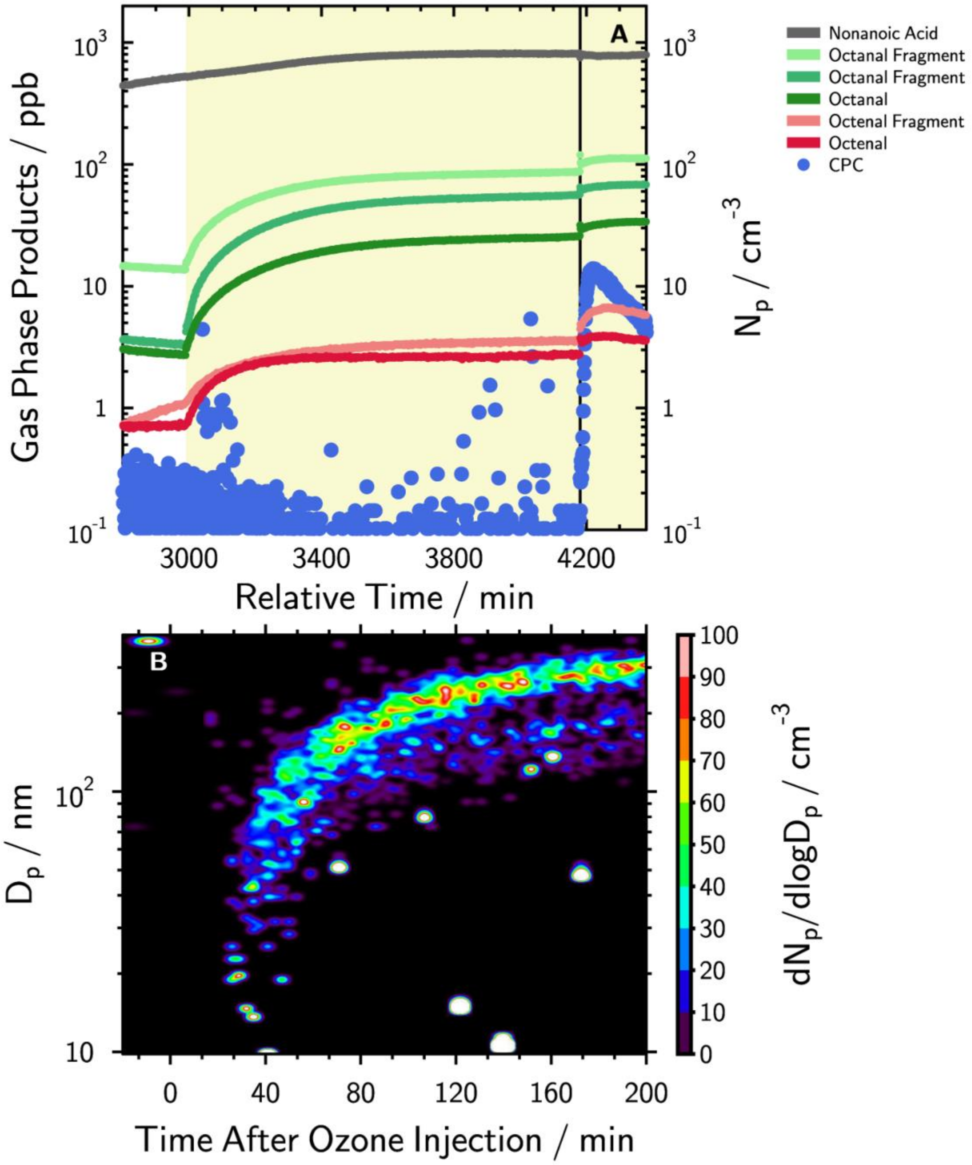

Figure S4: A) Concentration of volatile organic compounds (VOCs) using the H_3O^+ ionization mode and secondary organic aerosol (SOA) particles in a chamber experiment as a function of time, *t* in which nonanoic acid was present at an air-water interface. B) SOA particle size distribution, $dN_p/d\log D_p$, as a function of *t* after ozone injection for the nucleation event shown in panel A. All other lines and shadings are the same as in Fig. S2.

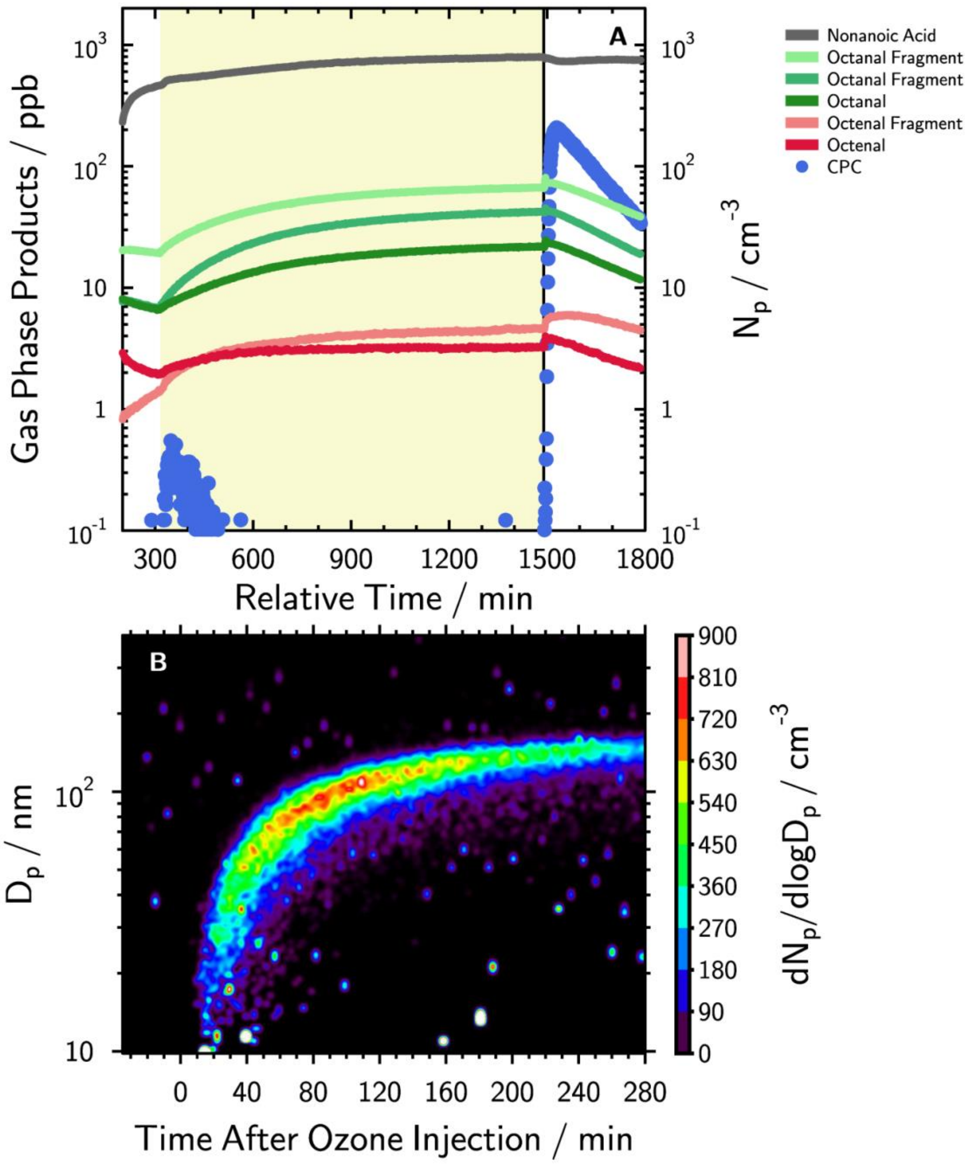

Figure S5: A) Concentration of volatile organic compounds (VOCs) using the H_3O^+ ionization mode and secondary organic aerosol (SOA) particles in a chamber experiment as a function of time, *t* in which nonanoic acid was present at an air-aqueous solution interface of humic acid and water. B) SOA particle size distribution, $dN_p/d\log D_p$, as a function of *t* after ozone injection for the nucleation event shown in panel A). All other lines and shadings are the same as in Fig. S2.

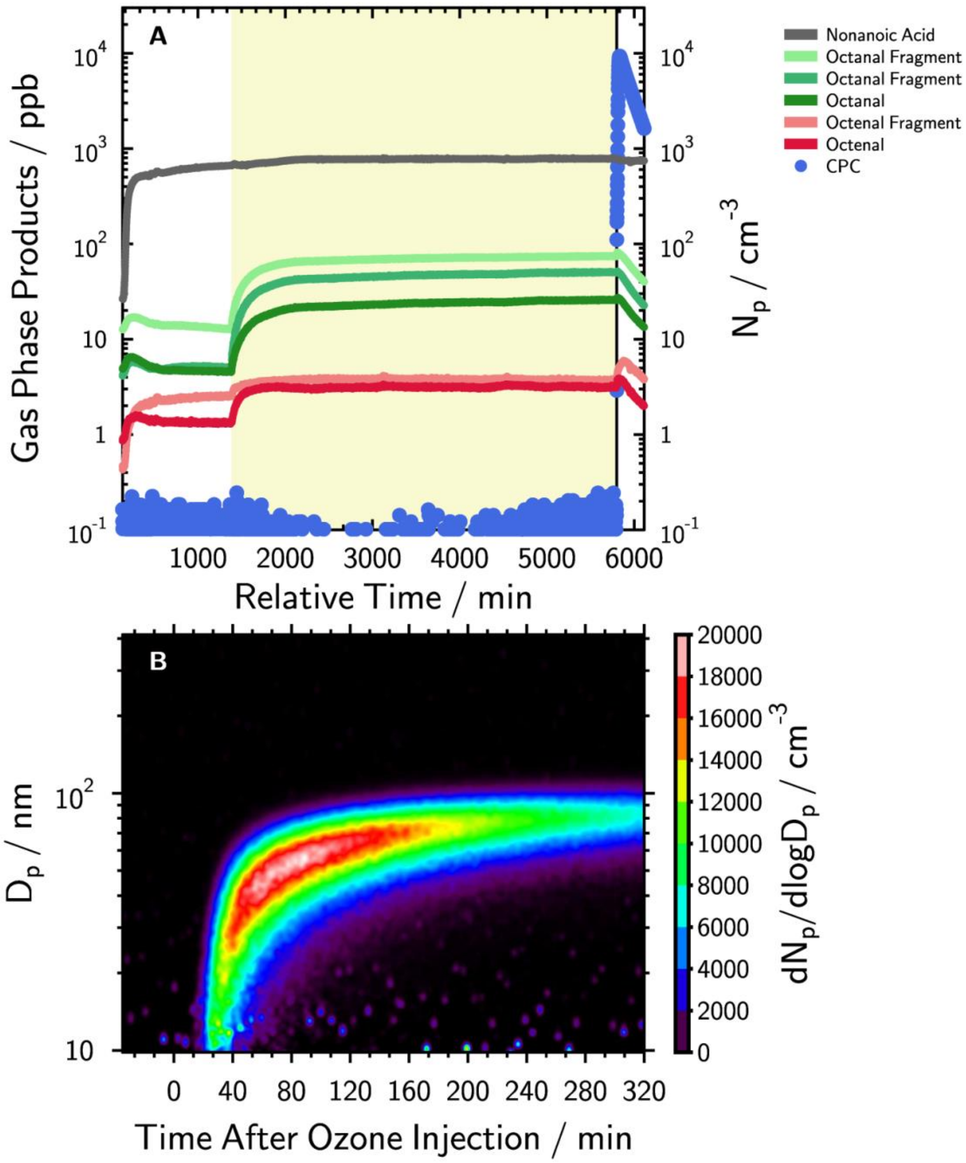

Figure S6: A) Concentration of volatile organic compounds (VOCs) using the H_3O^+ ionization mode and secondary organic aerosol (SOA) particles in a chamber experiment as a function of time, *t* in which nonanoic acid was present at an air-aqueous solution interface of humic acid and water. B) SOA particle size distribution, $dN_p/d\log D_p$, as a function *t* after ozone injection for the nucleation event shown in panel A). All other lines and shadings are the same as in Fig. S2.

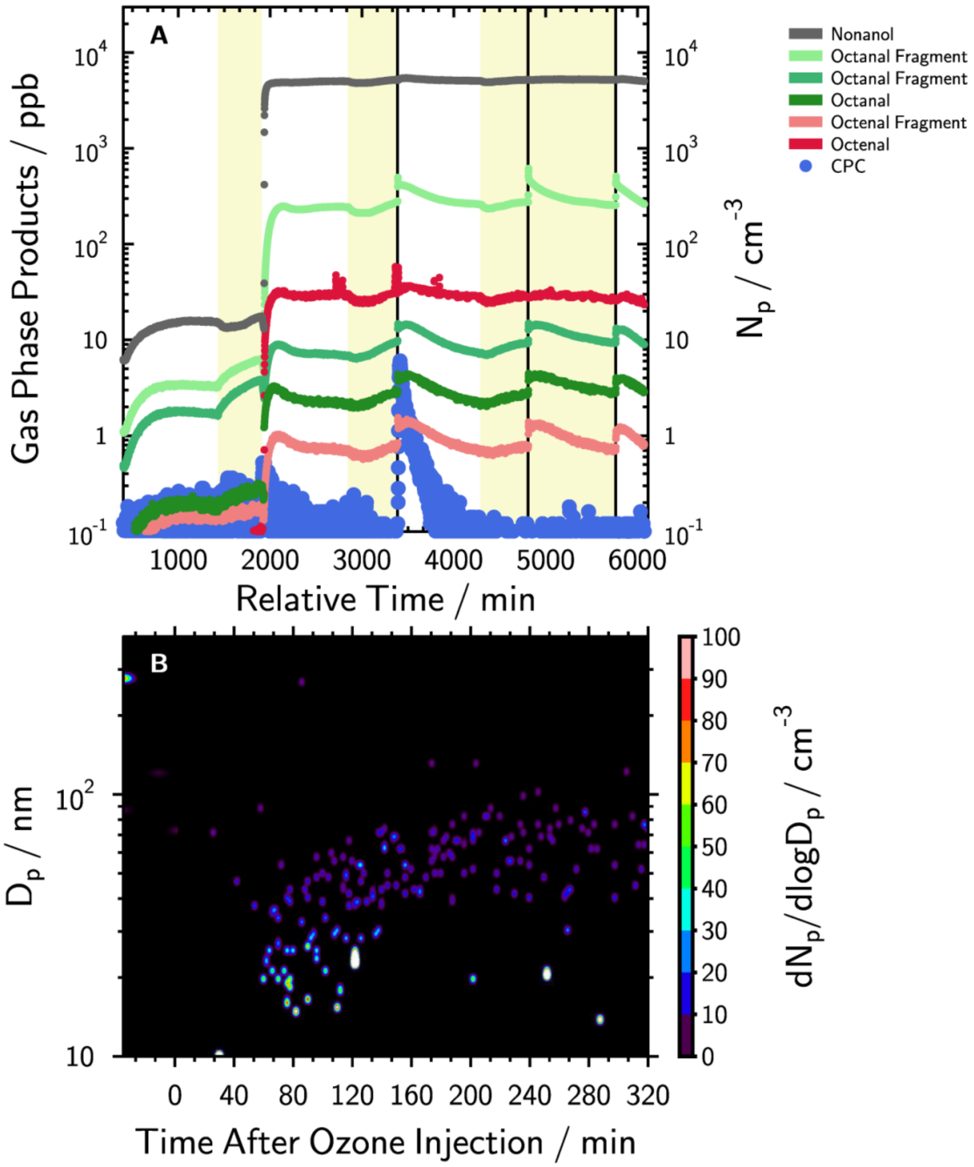

Figure S7: A) Concentration of volatile organic compounds (VOCs) using the H_3O^+ ionization mode and secondary organic aerosol (SOA) particles in a chamber experiment as a function of time, *t* in which nonanol was present at an air-water interface. Ozone was injected at three different times indicated by solid vertical lines. B) SOA particle size distribution, $dN_p/dlogD_p$, as a function *t* after the first ozone injection for the nucleation event shown in panel A). All other lines and shadings are the same as in Fig. S2.

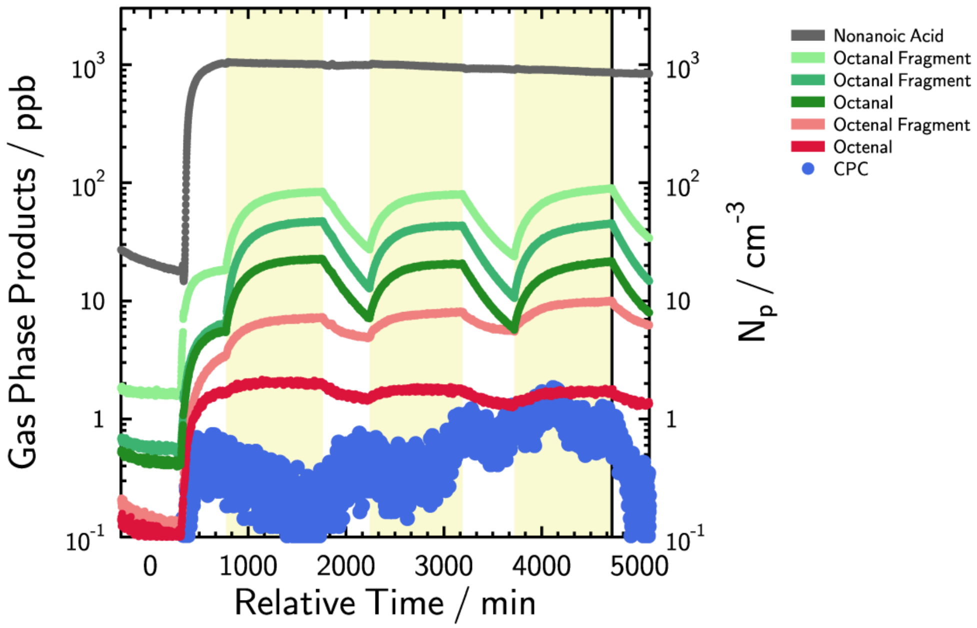

Figure S8: Concentration of volatile organic compounds (VOCs) using the H_3O^+ ionization mode and secondary organic aerosol (SOA) particles in a chamber experiment as a function of time, *t* in which nonanoic acid was present in the gas phase. The solid vertical line indicated ozone injection up to 50 ppb. All other lines and shadings are the same as in Fig. S2.

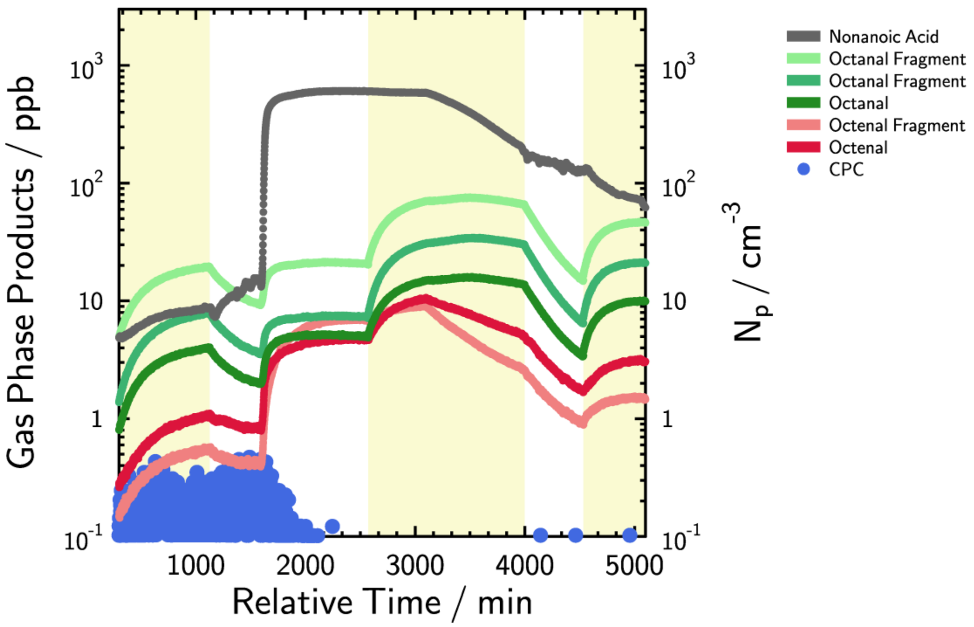

Figure S9: Concentration of volatile organic compounds (VOCs) using the H_3O^+ ionization mode and secondary organic aerosol (SOA) particles in a chamber experiment as a function of time, *t* in which nonanoic acid was present in the gas phase. All other lines and shadings are the same as in Fig. S2.

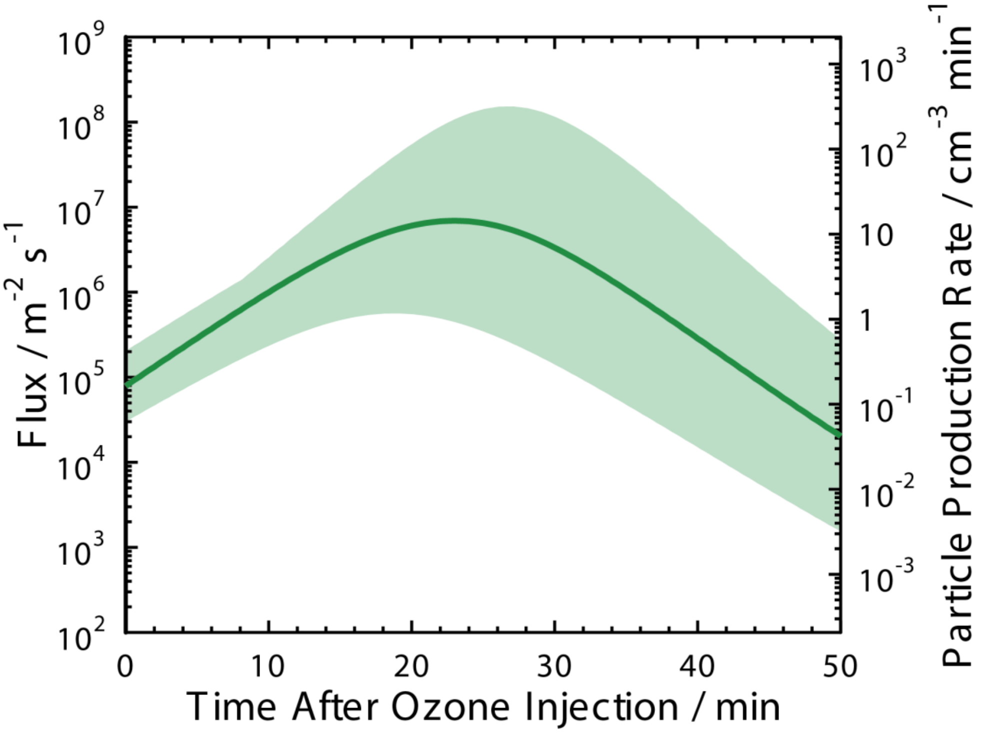

Figure S10: Experimentally derived flux and particle production rates of secondary organic aerosol (SOA) particles derived from sigmoidal fits of the observed particle concentrations presented in Fig. S2-S5. The solid line and shading indicate the mean and range of values as a function of time, *t*.

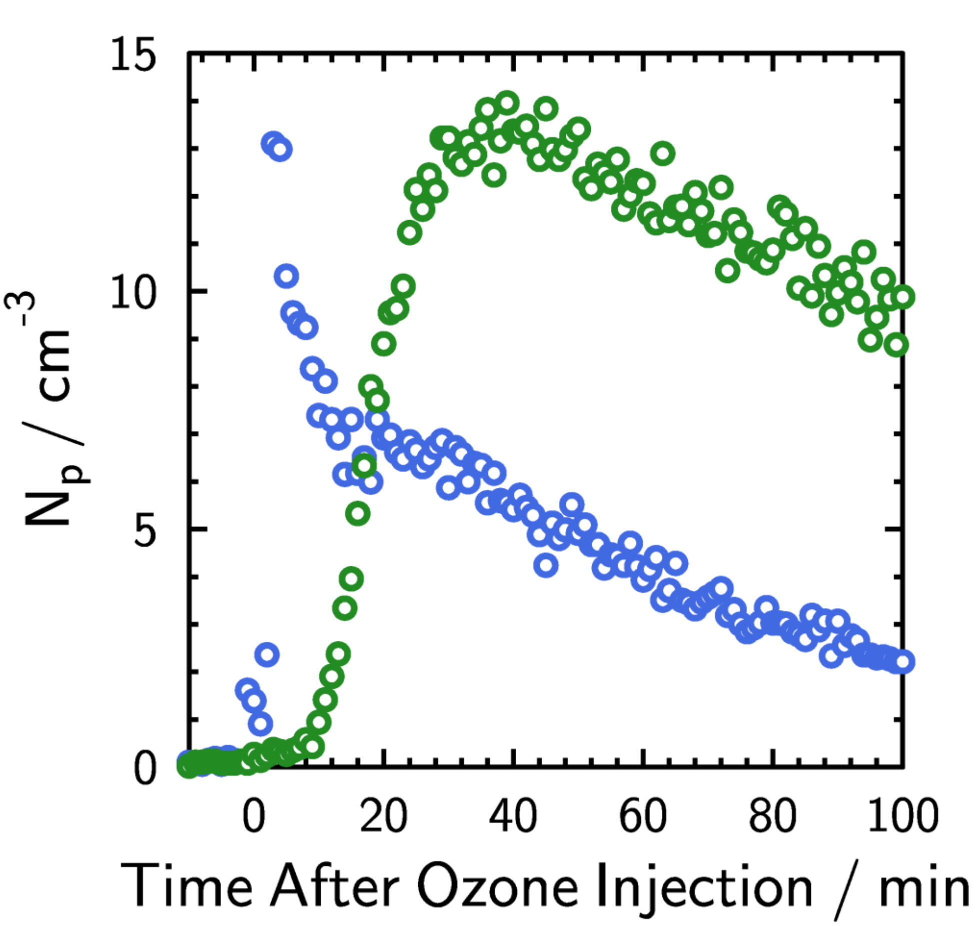

Figure S11: Aerosol particle concentrations in two different experiments as a function of time after ozone injection. The green symbols indicate new particle formation that occurred subsequent to UV irradiation of nonanoic acid at the air-water interface. The blue symbols indicate an immediate particle increase when ozone, produced by corona discharge, was injected into a chamber which contained water only.

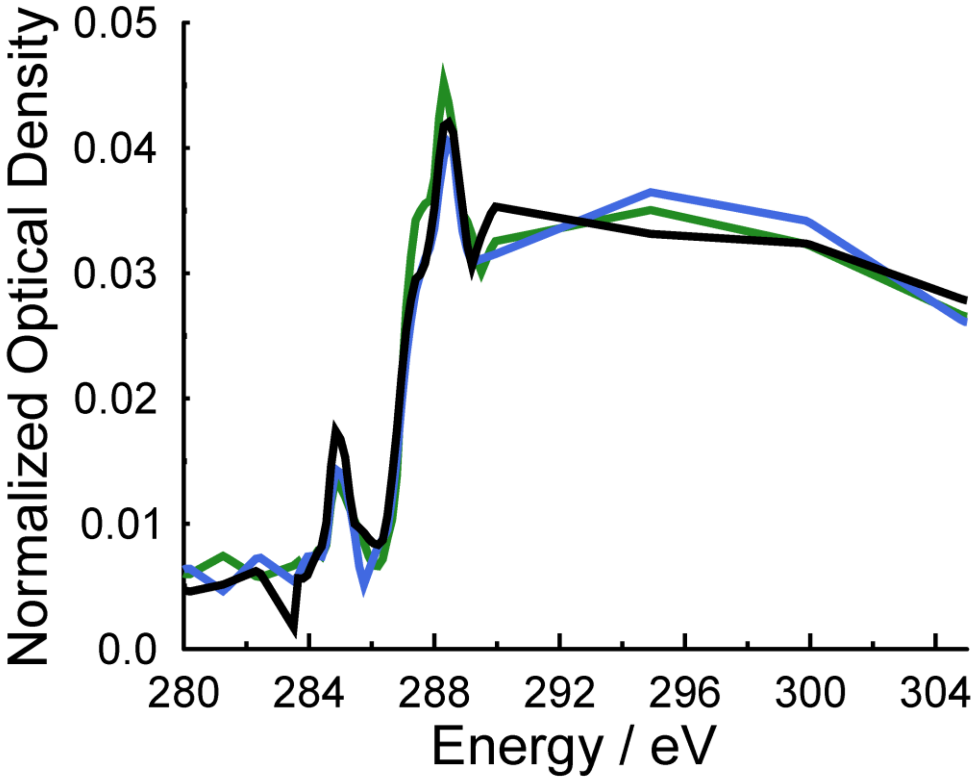

Figure S12: Normalized optical density as a function of photon energy of the same particles irradiated multiple times to investigate X-ray beam damage. Green, blue and black spectra are consecutive measurements and indicate low, medium and high levels of X-ray exposure. Normalization was performed by dividing the spectra by its total integrated area.
